# Prognostic Nomogram and Therapeutic Option of Cancer-Specific Death in the Patients with Metachronous Second Primary Lung Cancer

**DOI:** 10.1155/2022/2819798

**Published:** 2022-01-18

**Authors:** Jiahui Wang, Zhen Chen, Daokui Xia, Xinyu Song, Zhigang Hu

**Affiliations:** ^1^Department of Respiratory and Critical Care Medicine, The First College of Clinical Medicine science, China Three Gorges University, Yichang 443003, China; ^2^Department of Respiratory and Critical Care Medicine, Yichang Central People's Hospital, Yichang 443003, China; ^3^Department of Thoracic Surgery, The First College of Clinical Medicine Science, China Three Gorges University, Yichang 443003, China

## Abstract

With the increase of long-term primary lung cancer survivors, studies focused on metachronous second primary lung cancer (SPLC) have become very urgent. This study aimed to develop a prognostic nomogram and determine therapeutic options of cancer-specific death for patients with metachronous SPLC with and without the competing risk of other-specific death. Study population came from the SEER-18 database between 2006 and 2016. According to the clinical practice guideline of SPLC, the interval time of IPLC and metachronous SPLC was set to 4 years. We constructed nomograms with Lasso + Cox regression model and competing risk model to predict the prognosis and identify therapeutic options of metachronous SPLC patients with the assessment of model performance by the C-index, calibration plot, and decision curve analysis. In addition, two subgroup analyses stratified by histology and tumor size were used to better select therapeutic options for a certain population. 1300 patients with metachronous SPLC were incorporated in this study with 50.1% of the 5-year cumulative incidence in cancer-specific death. Compared with Lasso + Cox regression analysis, competing risk analysis harbored a higher C-index (0.811 vs. 0.76) and better net benefit in predicting cancer-specific death of metachronous SPLC. Two statistical analyses suggested that surgery alone was a preferentially therapeutic option of metachronous SPLC, whereas the effect of surgery + radiation in treating metachronous SPLC was similar to radiation alone. Subgroup analyses indicated that patients with metachronous SPLC were considered receiving different therapeutic options in different histology and tumor size but preferred to receive surgical treatment as the first choice. For primary lung cancer survivors, aggressive surgical treatment was the first-line selection of metachronous SPLC, followed by radiation alone, surgery + radiation, and no surgery + radiation.

## 1. Introduction

Because of air pollution and tobacco use in the past decades, lung cancer has become one of the most common carcinomas with high morbidity and mortality worldwide [1, 2]. However, the survival time of lung cancer is gradually prolonged due to the advancements in cancer treatment and the CT screen of early lung cancer. Low volume CT screening was associated with significantly lower risk of lung cancer death compared with chest radiography [3] and no screening [4]. Therefore, studies on metachronous second primary lung cancer (SPLC) of long-term lung cancer survivors are urgently needed. Current studies reported approximately 1% to 2% annual incidence of lung cancer among patients with initial primary lung cancer (IPLC), which was fourfold to sixfold higher than those with no history of lung cancer [5–7]. The cumulative risk of SPLC can reach 20.2% at 10 years postsurgery (95% confidence interval (CI): 15.3–23.2), and 25.2% (95% CI: 19.4–31.3) at 14 years postsurgery [8]. Prognostic risk factors of SPLC need to become the focus of clinicians' research.

Previous studies reported some independent risk factors of overall survival and cancer-specific death among patients with SPLC, including tumor stage [9], tumor size [10, 11], lymph node metastasis [12], and surgery [13–15]. Compared with IPLC, SPLC treatment needs to take into full consideration the influence of surgery on IPLC. Most studies about overall survival and cancer-specific death among patients with SPLC are based on the Kaplan–Meier method and multivariate Cox proportional regression analysis [9–15]. For old patients with SPLC, the competing risks of cardiovascular and cerebrovascular diseases are inevitable. Competing risk regression analysis is more available to determine the independent risk factors of cancer-specific death among the patients with SPLC in the presence of other-specific death. In addition, a competing risk nomogram can provide a graph of the mathematical model to predict special endpoints on the basis of various biological and clinical variables with relation to cancer-specific death. There is no published study about competing risk analysis and nomogram of cancer-specific death among patients with metachronous SPLC as yet.

Our first study aim is to obtain the 1-year and 5-year cumulative incidence of cancer-specific death and other-specific death among patients with metachronous SPLC by using competing risk regression analysis. Our second study aim is to determine independent prognostic factors of cancer-specific death with and without the competing risk of other-specific death. Subsequently, we construct nomograms with the Cox regression model and competing risk model to predict the prognosis of metachronous SPLC patients and assess model performance by the C-index, calibration plot, and decision curve analysis. In addition, subgroup analysis is performed to better select therapeutic options for a certain population.

## 2. Methods

### 2.1. Study Population

Surveillance, Epidemiology, and End Results (SEER) database is a representative population-based database of the National Cancer Institute, which covers 18 states across the United States and approximately 28% of the U.S. population with the strictest data-quality indicators and consistent criteria [16]. In our study, we extracted the data about metachronous SPLC between 2006 and 2016 from the SEER-18 database(SEER ID: huzg) released in April 2019 using the SEER *∗* Stat software (Version 8.3.4, https://seer.cancer/gov/seerstat/). The definition of metachronous SPLC was proposed by Martini and Melamed [17]: (1) interval time between IPLC and SPLC ≥2 years; (2) different histology between IPLC and SPLC; (3) When histology between IPLC and SPLC was the same, IPLC and SPLC must origin in situ or locate in different lobes or segments, with no positive intervening lymph nodes and no evidence of metastasis. Because an interval time of 2 to 4 years was considered a gray area [18], the interval time of IPLC and SPLC was set to 4 years in our study. Patients with unknown histological types in IPLC and SPLC were excluded. According to the International Classification of Diseases of Oncology (3rd edition), the histology of IPLC and SPLC was crudely classified into five groups: small cell carcinoma, large cell carcinoma, squamous cell carcinoma, adenocarcinoma, and others. Because partial patients came from 2004 to 2010, American Joint Committee on Cancer sixth Edition staging system was used to perform tumor stage of IPLC and SPLC in our study. No other lung cancer was diagnosed before the IPLC and after SPLC. Detailed surgical options of IPLC and SPLC were not included in the recently released SEER-18 database. Therefore, surgical options of metachronous SPLC were crudely classified into four groups (no vs. surgery alone vs. surgery + radiation vs. radiation alone) according to” Radiation sequence with surgery”, “Radiation recode,” and “Reason no cancer-directed surgery.” Tumor size (≤2 cm) was found to be an independent risk factor of metachronous SPLC [10, 13, 15]; thus we have taken the same classification in terms of tumor size (≤2 cm vs > 2 cm). The survival status of patients with metachronous SPLC was classified into alive, cancer-specific death, and other-specific death with the final follow-up time of Dec 2016. In addition, we collected some patients' demographics, basic characteristics of IPLC and metachronous SPLC as potential confounding factors, including age (SPLC), sex, race, marital status at diagnosis (SPLC), laterality (IPLC), laterality (SPLC), primary Site (IPLC), primary Site (SPLC), AJCC *T* 6th ed (SPLC), AJCC N 6th ed (SPLC), AJCC *M* 6th ed (SPLC), chemotherapy record (IPLC), chemotherapy record (SPLC), SEER Combined Summary Stage (SPLC), and survival time (SPLC).

### 2.2. Ethics

There were no patients involved in the recruitment and conduct of our study. Therefore, our study was deemed exempt for review by the Institutional Review Board at China, Three Gorges University.

### 2.3. Statistical Analysis

Our study population was divided into three groups according to survival status. Demographic and clinical characteristics of the three groups were summarized with counts and frequencies, and tested with chi-square tests. The normality of distribution of the data was tested by chi-square goodness-of-fit and Kolmogorov–Smirnov tests. Cumulative incidence function (CIF) was used to estimate the probability of cancer-specific death and other-specific death during follow-up by using Fine and Gray's competing risk regression analysis. 23 variables were regarded as the potential risk factors of cancer-specific death in our study. For high-dimensional data, the least absolute shrinkage and selection operator (LASSO) method with 10-fold cross-validation is more available to select the useful predictive features from the primary data than conventional univariate regression analysis [19, 20]. Subsequently, multivariate Cox regression analysis with proportional hazard model was used to determine risk factors and develop prognostic nomogram of cancer-specific death. We checked the proportional hazards assumption by using statistical tests and graphical diagnostics on the base of the scaled Schoenfeld residuals. We also compared the difference of cancer-specific death stratified by each potential risk factor through a competing risk analysis with proportional subdistribution hazard model. Before multivariate competing risk analysis, we evaluated the problem of collinearity of all potential risk factors by testing tolerance and variance inflation factor. When the tolerance of each variable was less than 0.1 and variance inflation factor was greater than 5, this variable was considered omitted from this study [21]. Based on multivariate competing risk regression analysis with proportional subdistribution hazard model [22], we examined the independent risk factors of cancer-specific death and constructed a competing risk nomogram to predict 1-year and 5-year probabilities of cancer-specific death. Akaike information criterion and the Bayesian information criterion (BIC) were used for competing risk regression analysis. We estimated the discrimination performance of prognostic nomogram by Concordance index (C-index) values and graphical calibration by plotting the observed rates against the nomogram-predicted probabilities through a bootstrap cross-validation approach with 1000 resamples [22]. The decision curve analysis for survival data with competing risk was used to evaluate the prediction model from the perspective of clinical consequences by calculating the net benefit [23]. When the prediction model produced a larger net benefit, the clinical use of decision curve analysis could effectively discriminate false-positive and false-negative results. In addition, we performed two subgroup analyses of tumor size and histology to better characterize the prognostic differences between chemotherapy and radiation/surgery. The subdistribution hazard ratio (sHR) with 95% CIs was estimated to evaluate the differences, and a two-tailed *P* < 0.05 was considered statistically significant.

All statistical analyses were performed by using R version 3.4.2 software and Stata 14. Cumulative incidence function and the construction of competing risk nomogram were based on the following R packages: “cmprsk”, “rms”, and “mstate”. R package “riskRegression” and “pec” were used to construct calibration plot [22].

## 3. Results

### 3.1. Patient Characteristics

**Figure 1:**
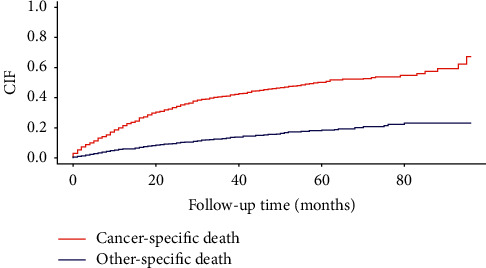
The cumulative incidences of cancer-specific death and other-specific death among metachronous SPLC during 10-year follow-up.

A total of 1300 metachronous SPLC patients with IPLC and an interval time of ≥4 years were incorporated in the study according to our selection criteria. The median age at diagnosis of metachronous SPLC was 73 years (interquartile range (IQR): 67–99 years). The majority of the study population was female (58.2%) and White (81.5%). The upper lobe (52.8%) was the most common site of metachronous SPLC, followed by the lower lobe (36.8%) and other areas (10.4%). Similar to IPLC, the main histology types of metachronous SPLC were adenocarcinoma (55.3%) and squamous cell carcinoma (24.5%). Among the total number of patients, 75.2% showed different histologies for IPLC and metachronous SPLC. There was 62.4% of metachronous SPLC with stage I. A total of 1014 patients (78%) had no positive lymph node involvement. The median interval time between IPLC and metachronous SPLC was 69 months (IQR: 57–86 months). The most common treatment of patients with metachronous SPLC was surgery alone (39.6%), followed by radiation alone (30.3%), no surgery/radiation (20.3%), and surgery + radiation (9.8%). Until Dec 2016, 582 patients of metachronous SPLC were alive, with 538 cancer-specific deaths and 180 other-specific deaths. The median follow-up time of individuals with metachronous SPLC was 30 months (IQR: 19–46 months) in the alive group, followed by the other-specific death group (17 months, IQR:7.0–31.2 months), and the cancer-specific death group (12 months, IQR:5.0–24 months). Detailed demographic and clinical characteristics of three survival status groups were shown in Table 1. The overall 1-year, 3-year, and 5-year mortality of metachronous SPLC were 25.2%, 52.7%, and 68.2%, respectively. When considering the competing risk of other-specific death, the 1-year, 3-year, and 5-year cumulative incidence of cancer-specific death were 19.8%, 40.3%, and 50.1%, respectively (see Figure 1).

### 3.2. The Construction and Assessment of Prognostic Nomogram of Cancer-Specific Death without Regard to Competing Risk

Lasso analysis with Cox regression model observed 16 potential risk factors of cancer-specific death with 0.84 of the area under the receiver operating characteristic curve (see Figure S1). Multivariate Cox regression analysis further found that the independent risk factors of cancer-specific death comprised seven variables, including age, sex, marital status, histology (SPLC), AJCC N (6 eth, SPLC), AJCC *T* (6 eth, SPLC), and radiation/surgery (SPLC) (see Table S1). Unmarried old male with small cell carcinoma and mediastinal lymph node involvement harbored worse clinical outcomes. Based on these risk factors, we constructed a prognostic nomogram of cancer-specific death with the Cox regression model (see Figure S2). Histology (SPLC) and AJCC N (6 eth, SPLC) were two main risk factors of cancer-specific death. The C-index of this nomogram was 0.736 (95% CI: 0.691–0.781) with an acceptable calibrated plot (see Figure S3). We also performed decision curve analysis to assess the clinical net benefit of the prognostic nomogram, which demonstrated a wide range of thresholds for predicting 1-year probability (9%–76%) and 5-year probability (29%–91%) of cancer-specific death (see Figure S4).

### 3.3. The Construction and Assessment of Prognostic Nomogram of Cancer-Specific Death in the Present of Competing Risk

**Figure 2:**
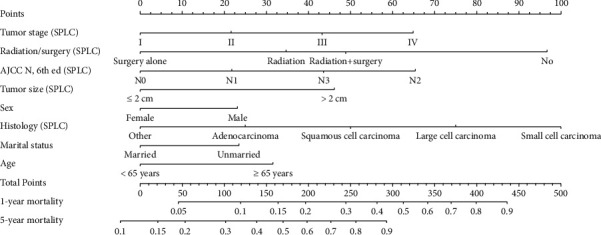
The prognostic nomogram for predicting the 1-year and 5-year probability of cancer-specific death among metachronous SPLC by using competing risk model. Total point values were independently calculated for each cause of death and then applied to the corresponding probability scale at the bottom of each.

**Figure 3:**
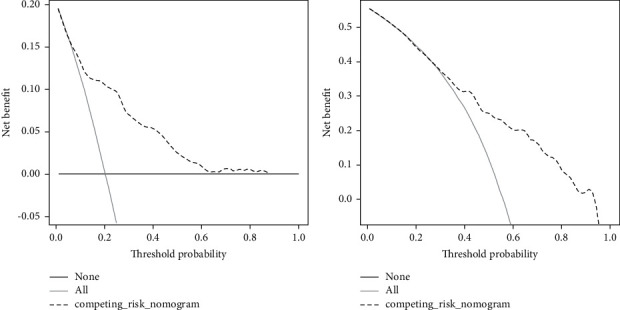
Decision curve analysis for cancer-specific death model among metachronous SPLC by using competing risk model. (a) 1-year probability of cancer-specific death; (b) 5-year probability of cancer-specific death.

Univariate and multivariate competing risk regression analyses (see Table 2) suggested the independent risk factors of cancer-specific death as follow: age, sex, marital status, tumor size, histology (SPLC), AJCC N (6 eth, SPLC), tumor stage (SPLC), and radiation/surgery (SPLC). Surgery alone (no radiation/surgery vs surgery alone, sHR = 0.41, 95%CI: 0.3–0.55, *P* < 0.001) and radiation alone (no radiation/surgery vs radiation alone, sHR = 0.60, 95% CI: 0.46–0.78, *P* < 0.001) were associated with significantly lower risk of cancer-specific death compared with no radiation/surgery. We integrated eight independent risk factors to develop the prognostic competing risk nomogram of 1-year and 5-year probability of cancer-specific death. The C-index value was 0.811 (95% CI: 0.805–0.817) with an acceptable calibrated plot (see Figure S5), which seemed to be higher than that of prognostic nomogram with Cox regression model (C-index = 0.736, 95% CI: 0.691–0.781). Through this competing risk nomogram, we visually estimated the contribution of each included variable in cancer-specific death (see Figure 2). Of all eight variables, histology of metachronous SPLC harbored the biggest contribution to cancer-specific death, especially small cell carcinoma. In terms of tumor stage, stage IV metachronous SPLC was associated with the highest risk of cancer-specific death, followed by stage III, stage II, and stage I. At the aspect of AJCC *N*, 6th ed (SPLC), the mediastinal involvement (*N*2) seemingly had the significantly bigger contribution to cancer-specific death compared with N0. By calculating the sum of points of the eight variables, we predicted the 1-year and 5-year probabilities of cancer-specific death among patients with metachronous SPLC. Moreover, decision curve analysis demonstrated that our competing risk nomogram produced clinical net benefit in all ranges for predicting 1-year probability of cancer-specific death and in a wide range of thresholds (27%–93%) for predicting 5-year probability (see Figure 3).

### 3.4. Subgroup Analyses of Histology and Tumor Size in Competing Risk Analyses

For nonsmall cell carcinoma, surgery alone, radiation alone, and radiation + surgery had significant therapeutic effects in cancer-specific death compared with no radiation/surgery (see Table 3). However, patients with small cell carcinoma might consider receiving surgery alone (no radiation/surgery vs. surgery alone, sHR = 0.28, 95% CI: 0.11–0.76, *P*=0.012) and radiation alone (no radiation/surgery vs radiation alone, sHR = 0.41, 95% CI: 0.18–0.90, *P*=0.026), but not surgery + radiation (no radiation/surgery vs. surgery + radiation, sHR = 0.85, 95% CI: 0.28–2.55, *P*=0.769).

The authors also stratified the patients by tumor size of metachronous SPLC (≤2 cm vs >2 cm). Chemotherapy had no significant impact on cancer-specific death whether tumor size was less than 2 cm or larger than 2 cm. When tumor size was less than 2 cm, only surgery alone was associated with a lower risk of cancer-specific death than no surgery + radiation (no radiation/surgery vs. surgery alone, sHR = 0.52, 95% CI: 0.31–0.87, *P*=0.013). When tumor size was more than 2 cm, the clinical benefit of cancer-specific death was significant in the surgery alone (no radiation/surgery vs. surgery alone, sHR = 0.33, 95% CI: 0.23–0.48, *P* < 0.001) and radiation alone (no radiation/surgery vs. radiation alone, sHR = 0.63, 95% CI: 0.47–0.86, *P*=0.003) groups compared with the no radiation/surgery group.

## 4. Discussions

This study estimated the cumulative incidence of cancer-specific death in patients with metachronous SPLC, differentiated the influence of other-specific death, and determined potential risk factors of cancer-specific death. The 5-year cumulative incidence of cancer-specific death and other-specific death were 50.1% and 18.1%, respectively. Our study also found that the C-index of competing risk model (0.811, 95% CI:0.805–0.817) was superior to that of Cox regression model (0.736, 95% CI: 0.691–0.781). In addition, our subgroup analyses indicated that the patients of metachronous SPLC with small cell carcinoma and nonsmall cell carcinoma initially preferred to receive surgery alone. When tumor size was less than 2 cm, surgery alone was considered as the main therapeutic option of metachronous SPLC. When tumor size was more than 2 cm, surgery alone and radiation alone were also proposed as therapeutic options for metachronous SPLC along with the preferred method of surgery alone.

Through multivariate competing risk analysis and nomogram points, the authors found some independent risk factors of cancer-specific death among patients with metachronous SPLC. In terms of histology (SPLC), small cell carcinoma seemingly had the biggest contribution to cancer-specific death, followed by large cell carcinoma, squamous cell carcinoma, other, and adenocarcinoma. The vast majority of existing studies focused on nonsmall cell carcinoma of metachronous SPLC [13, 24] and the comparison of operational modes of early SPLC [15, 25, 26]. Our study suggested that surgery alone and radiation alone might be therapeutic options for metachronous SPLC with small cell carcinoma. For metachronous SPLC with nonsmall cell carcinoma, the effect of surgery alone on cancer-specific death seemed to be superior to those of other therapeutic options, especially when tumor size was ≥2 cm. Our study also showed that radiation alone had significant clinical benefit compared with no radiation/surgery (no radiation/surgery vs radiation alone, sHR = 0.63, 95% CI: 0.47–0.86, *P*=0.003) when tumor size was larger than 2 cm. Taioli and the colleagues conducted the first study on the effect of radiation and surgery on SPLC survival [27]. The results obtained by multivariate Cox proportional hazards model and propensity score analysis showed that patients with SPLC who underwent surgery alone demonstrated significantly longer survival than those who underwent radiation alone and no radiation/surgery. Another propensity score analysis from a Chinese population showed that the 5-year overall and progression-free survival of metachronous SPLC with surgical treatment were significantly better than those without surgery [14]. No published study has determined the effect of radiation + surgery on cancer-specific death by using competing risk analysis. Two other important risk factors of CSD, namely, tumor stage and lymph node metastasis, were mentioned in our study (see Figure 2). In multivariate competing risk analysis, stage I was associated with significantly lower cancer-specific mortality than stage III (stage I vs stage III, sHR = 1.81, 95% CI: 1.03–3.19, *P*=0.039) and stage IV (stage I vs stage IV, sHR = 2.17, 95% CI: 1.23–4.18, *P*=0.020). Three previous studies [9, 10, 28] also reported that TNM stage was an independent risk factor of survival in metachronous SPLC, especially stage I. Lee and the colleagues even declared that TNM stage is the most important determinant of survival in metachronous lung cancer [9]. Our study found that mediastinal involvement (*N*2) of metachronous SPLC was associated with significantly worse survival than *N*0 (*N*0 vs. *N*2, sHR = 1.75, 95% CI: 1.15–2.87, *P* < 0.01), which was similar to the study results of Riquet et al. [12] and Yang et al. [26]. By multivariate Cox regression analysis, tumor size (≤2 cm) was regarded as an important risk factor of overall and cancer-specific survival in some studies [10, 15, 24, 26]. In addition, tumor size (≤2 cm) was an important reference for operation modes and can be a basis for the performance of sublobar resection [24, 26]. Our competing risk analysis suggested that a smaller tumor size was associated with a lower risk of cancer-specific death (≤2 cm vs >2 cm, sHR = 1.39, 95% CI: 1.12–1.73, *P*=0.003). In subgroup analysis on the basis of tumor size, only surgery alone harbored a significant effect on cancer-specific mortality when tumor size was less than 2 cm. When tumor size was larger than 2 cm, surgery alone and radiation alone could obtain significant clinical benefits of cancer-specific death compared with no radiation/surgery. There are no published studies about the comparison between the effects of radiation and surgery on cancer-specific death of metachronous SPLC stratified by tumor size. The competing risk nomagram indicated that the other three risk factors (sex, age, marital status) had relatively little contributions to cancer-specific death. Our study also observed that histology between IPLC and metachronous SPLC (different vs same) and surgery/radiation of IPLC (no vs. yes) had no significant effect on cancer-specific death, which were similar to the previous studies [12, 16, 26].

Our study had the following strengths. We included 1300 patients from 18 states across the United States; this was approximately 28% of the U.S. population. Our sample size effectively decreased the selective bias of a small sample size and single-center study. We examined the independent risk factors of cancer-specific death by simultaneously using Cox regression analysis and competing risk regression analysis. We also developed and compared prognostic nomograms to guide clinicians' risk evaluations for cancer-specific mortality among patients with metachronous SPLC. In addition, our subgroup competing risk analyses provided important references for therapeutic options in cases with different histologies and for selecting between radiation and surgery in metachronous SPLC cases with different tumor sizes.

Certainly, our study also had some limitations. Firstly, some important details, such as smoking status and cardiopulmonary function, were not included in the SEER database, which potentially affected our study results. Patients' clinical backgrounds might affect the selection of therapeutic options. Surgery selects the fittest patients. Radiation selects intermediary fit. Undocumented patient's characteristics are likely to result in selection bias. Secondly, the newly released SEER-18 did not reveal surgical treatments. The authors were not able to assess the advantages and disadvantages of surgical treatments in specific populations. Thirdly, the lack of external validation set potentially affected the confidence of our study results. Although competing risk analysis with proportional subdistribution hazards regression can obtain unbiased estimates of the risk of cancer-specific death, potential bias was still inevitable due to the retrospective nature of the study.

## 5. Conclusion

Our study provided the cumulative incidence of cancer-specific death among metachronous SPLC on the basis of a large-sample study population with considering the competing risk of other-cancer death. A prognostic nomogram with a competing risk model was constructed to predict the 1-year and 5-year probabilities of cancer-specific death with acceptable performance and decision curve analysis. Our subgroup competing risk analyses provided evidence-based references for therapeutic options of metachronous SPLC in specific populations. Future prospective studies and random controlled studies should be conducted to determine the choice of radiation and surgery in certain patients with metachronous SPLC.

## Figures and Tables

**Table 1:** The detailed demographic and clinical characteristics of study population.

	Survival status			*P*-value
	Alive	Cause-specific death	Other-specific death	
*N*	582	538	180	
Age(years)	72.0 (66.0–77.0)	74.0 (68.0–80.0)	73.0 (66.8–80.0)	<0.001

Age				0.062
<65 years	120 (20.6%)	82 (15.2%)	31 (17.2%)	
≥65 years	462 (79.4%)	456 (84.8%)	149 (82.8%)	

Sex				<0.001
Female	373 (64.1%)	281 (52.2%)	103 (57.2%)	
Male	209 (35.9%)	257 (47.8%)	77 (42.8%)	

Marital status				0.009
Unmarried	250 (43.0%)	264 (49.1%)	77 (42.8%)	
Married	332 (57.0%)	274 (50.9%)	103 (57.2%)	

Race				0.405
Other	37 (6.4%)	22 (4.1%)	7 (3.9%)	
Black	74 (12.7%)	77 (14.3%)	24 (13.3%)	
White	471 (80.9%)	439 (81.6%)	149 (82.8%)	

Laterality (SPLC)				0.672
Left	265 (45.5%)	231 (42.9%)	81 (45.0%)	
Right	317 (54.5%)	307 (57.1%)	99 (55.0%)	

Primary site (SPLC)				0.006
Main	7 (1.2%)	25 (4.6%)	2 (1.1%)	
Upper	302 (51.9%)	292 (54.3%)	92 (51.1%)	
Middle	49 (8.4%)	35 (6.5%)	17 (9.4%)	
Low	224 (38.5%)	186 (34.6%)	69 (38.3%)	

Histology (SPLC)				<0.001
Small cell carcinoma	17 (2.9%)	92 (17.1%)	10 (5.6%)	
Large cell carcinoma	16 (2.7%)	21 (3.9%)	6 (3.3%)	
Squamous cell carcinoma	116 (19.9%)	145 (27.0%)	58 (32.2%)	
Adenocarcinoma	381 (65.5%)	243 (45.2%)	95 (52.8%)	
Other	52 (8.9%)	37 (6.9%)	11 (6.1%)	

Tumor size (SPLC)				<0.001
≤2 cm	368 (63.2%)	192 (35.7%)	89 (49.4%)	
>2 cm	214 (36.8%)	346 (64.3%)	91 (50.6%)	

AJCC *N*, 6th ed (SPLC)				<0.001
*N*0	536 (92.1%)	333 (61.9%)	145 (80.6%)	
*N*1	13 (2.2%)	40 (7.4%)	14 (7.8%)	
*N*2	25 (4.3%)	121 (22.5%)	19 (10.6%)	
*N*3	8 (1.4%)	44 (8.2%)	2 (1.1%)	

AJCC *M*, 6th ed(SPLC)				<0.001
*M*0	555 (95.4%)	405 (75.3%)	157 (87.2%)	
*M*1	27 (4.6%)	133 (24.7%)	23 (12.8%)	

AJCC *T*, 6th ed(SPLC)				<0.001
*T*1	386 (66.3%)	229 (42.6%)	98 (54.4%)	
*T*2	103 (17.7%)	160 (29.7%)	43 (23.9%)	
*T*3	11 (1.9%)	26 (4.8%)	5 (2.8%)	
*T*4	82 (14.1%)	123 (22.9%)	34 (18.9%)	

Tumor stage (SPLC)				<0.001
I	450 (77.3%)	246 (45.7%)	115 (63.9%)	
II	24 (4.1%)	29 (5.4%)	14 (7.8%)	
III	81 (13.9%)	130 (24.2%)	28 (15.6%)	
IV	27 (4.6%)	133 (24.7%)	23 (12.8%)	

SEER combined summary Stage (SPLC)				<0.001
Localized	403 (69.2%)	220 (40.9%)	99 (55.0%)	
Regional	140 (24.1%)	150 (27.9%)	49 (27.2%)	
Distant	39 (6.7%)	168 (31.2%)	32 (17.8%)	

Chemotherapy (SPLC)				<0.001
Yes	100 (17.2%)	201 (37.4%)	42 (23.3%)	
No/unknown	482 (82.8%)	337 (62.6%)	138 (76.7%)	

Radiation/surgery (SPLC)				<0.001
Surgery alone	315 (54.1%)	134 (24.9%)	66 (36.7%)	
Radiation	173 (29.7%)	158 (29.4%)	63 (35.0%)	
Radiation + surgery	46 (7.9%)	65 (12.1%)	16 (8.9%)	
No	48 (8.2%)	181 (33.6%)	35 (19.4%)	

Primary site (IPLC)				0.004
Main	2 (0.3%)	10 (1.9%)	1 (0.6%)	
Upper	364 (62.5%)	345 (64.1%)	105 (58.3%)	
Middle	33 (5.7%)	51 (9.5%)	13 (7.2%)	
Low	183 (31.4%)	132 (24.5%)	61 (33.9%)	

Laterality (IPLC)				0.539
Left	268 (46.0%)	236 (43.9%)	75 (41.7%)	
Right	314 (54.0%)	302 (56.1%)	105 (58.3%)	

Histology (IPLC)				<0.001
Small cell carcinoma	29 (5.0%)	27 (5.0%)	5 (2.8%)	
Large cell carcinoma	24 (4.1%)	30 (5.6%)	17 (9.4%)	
Squamous cell carcinoma	108 (18.6%)	162 (30.1%)	52 (28.9%)	
Adenocarcinoma	367 (63.1%)	269 (50.0%)	93 (51.7%)	
Other	54 (9.3%)	50 (9.3%)	13 (7.2%)	

Histology of IPLC and SPLC				<0.001
Different	410 (70.4%)	433 (80.5%)	135 (75.0%)	
Same	172 (29.6%)	105 (19.5%)	45 (25.0%)	
Interval time of IPLC and SPLC(months)	70.0 (58.0–90.0)	68.0 (55.2–83.8)	67.0 (56.0–82.2)	0.003

Tumor stage (IPLC)				0.045
I	416 (77.0%)	382 (78.1%)	116 (69.0%)	
II	29 (5.4%)	28 (5.7%)	21 (12.5%)	
III	82 (15.2%)	67 (13.7%)	26 (15.5%)	
IV	13 (2.4%)	12 (2.5%)	5 (3.0%)	

Chemotherapy (IPLC)				0.397
Yes	149 (25.6%)	154 (28.6%)	44 (24.4%)	
No/unknown	433 (74.4%)	384 (71.4%)	136 (75.6%)	

Radiation/surgery (SPLC)				<0.001
Surgery alone	502 (86.3%)	414 (77.0%)	146 (81.1%)	
Radiation	27 (4.6%)	65 (12.1%)	12 (6.7%)	
Radiation + surgery	42 (7.2%)	45 (8.4%)	18 (10.0%)	
No	11 (1.9%)	14 (2.6%)	4 (2.2%)	

Follow-up time (months)	30.0 (19.0–46.0)	12.0 (5.0–24.0)	17.0 (7.0–31.2)	<0.001

*Note.* IPLC, initial primary lung cancer; SPLC, second primary lung cancer.

**Table 2:** Competing risk analyses of cancer cause death among patients with metachronous second primary lung cancer.

	Univariate analysis		Multivariate analysis	
	SHR	*P*	SHR	*P*
Age				
<65 years	Ref		Ref	
≥65 years	1.26 (1.01, 1.59)	0.049	1.32 (1.02, 1.72)	0.036

Sex				
Female	Ref		Ref	
Male	1.38 (1.16, 1.64)	<0.001	1.26 (1.03, 1.54)	0.024

Marital status				
Unmarried	Ref		Ref	
Married	0.77 (0.65, 0.92)	0.004	0.79 (0.65, 0.96)	0.021

Race				
White	Ref			
Black	1.29 (0.80, 2.08)	0.293		
Other	1.13(0.73,1.75)	0.574		

Laterality (SPLC)				
Left	Ref			
Right	1.01 (0.85, 1.20)	0.92		

Primary site(SPLC)				
Main	Ref		Ref	
Upper	0.36 (0.23, 0.56)	<0.001	0.85 (0.54,1.35)	0.5
Middle	0.26 (0.15, 0.44)	<0.001	0.53 (0.29, 0.96)	0.036
Low	0.32 (0.20, 0.50)	<0.001	0.75 (0.46,1.21)	0.233

Histology (SPLC)				
Small cell carcinoma	Ref		Ref	
Large cell carcinoma	0.39 (0.24, 0.65)	<0.001	0.85 (0.49, 1.45)	0.542
Squamous cell carcinoma	0.38 (0.29, 0.51)	<0.001	0.66 (0.47, 0.92)	0.015
Adenocarcinoma	0.24 (0.19, 0.31)	<0.001	0.49 (0.35, 0.67)	0.001
Other	0.28 (0.19, 0.42)	<0.001	0.48 (0.30, 0.79)	0.004

Tumor size(SPLC)				
≤2 cm	Ref		Ref	
>2 cm	2.23 (1.87, 2.66)	<0.001	1.39(1.12,1.73)	0.003

AJCC *N*, 6th ed (SPLC)				
*N*0	Ref		Ref	
*N*1	2.77 (1.94, 3.95)	<0.001	1.68 (1.06, 2.65)	0.027
*N*2	3.87 (3.07, 4.89)	<0.001	1.75 (1.19, 2.87)	0.004
*N*3	5.13 (3.73,7.91)	<0.001	1.70 (1.05, 2.74)	0.029

AJCC *T*, 6th ed(SPLC)				
*T*1	Ref		Ref	
*T*2	1.88 (1.54, 2.31)	<0.001	1.44 (1.17, 1.76)	<0.001
*T*3	2.68 (1.69, 4.26)	<0.001	1.36 (0.90, 2.36)	0.142
*T*4	1.96 (1.55, 2.49)	<0.001	1.07 (0.82, 1.41)	0.60

Tumor stage (SPLC)				
I	Ref		Ref	
II	1.71(1.13, 2.99)	0.011	1.34 (0.68, 2.62)	0.399
III	2.46 (1.98, 3.06)	<0.001	1.81 (1.03, 3.19)	0.039
IV	4.06 (3.18, 5.19)	<0.001	2.17 (1.23, 4.18)	0.020

SEER combined summary stage (SPLC)				
Localized	Ref		Ref	
Regional	1.71 (1.39, 2.10)	<0.001	0.75 (0.47, 1.18)	0.216
Distant	3.85 (3.07, 4.83)	<0.001	0.96 (0.51, 1.78)	0.887

Chemotherapy (SPLC)				
No/unknown	Ref		Ref	
Yes	2.23 (1.87, 2.67)	<0.001	0.85 (0.65, 1.13)	0.24

Radiation/surgery (SPLC)				
No	Ref		Ref	
Surgery alone	0.21 (0.17, 0.27)	<0.001	0.41 (0.30, 0.55)	<0.001
Radiation + surgery	0.57 (0.42, 0.76)	<0.001	0.68 (0.50, 0.94)	0.017
Radiation alone	0.45 (0.36, 0.57)	<0.001	0.60 (0.46, 0.78)	0.001

Primary site (IPLC)				
Main	Ref		Ref	
Upper	0.33 (0.19, 0.60)	<0.001	0.82 (0.37, 1.82)	0.623
Middle	0.43 (0.23, 0.80)	<0.001	1.0 (0.43, 2.34)	0.989
Low	0.28 (0.15, 0.50)	<0.001	0.66 (0.29,1.48)	0.315

Laterality (IPLC)				
Left	Ref			
Right	1.08 (0.97, 1.43)	0.391		

Histology (IPLC)				
Small cell carcinoma	Ref			
Large cell carcinoma	0.82 (0.46, 1.46)	0.494		
Squamous cell carcinoma	1.08 (0.69, 1.69)	0.725		
Adenocarcinoma	0.66 (0.43, 1.02)	0.059		
Other	0.77 (0.46, 1.29)	0.328		

Histology of IPLC and SPLC				
Different	Ref		Ref	
Same	0.66 (0.53, 0.81)	0.001	1.09 (0.87, 1.38)	0.459

Tumor stage (IPLC)				
I	Ref			
II	0.81 (0.55, 1.99)	0.291		
III	0.85 (0.65, 1.12)	0.258		
IV	0.96 (0.53, 1.74)	0.889		

Interval time of IPLC and SPLC	1.0 (0.995, 1.004)	0.969		

Chemotherapy (IPLC)	Ref			
No/unknown				
Yes	1.19(0.97,1.43)	0.068		

Radiation/surgery(IPLC)				
No	Ref			
Surgery alone	0.80(0.43,1.50)	0.487		
Radiation	0.91(0.46,1.82)	0.794		
Radiation + surgery	1.77(0.90,3.43)	0.098		

*Note.* SPLC, second primary lung cancer.

**Table 3:** Sub-group competing risk analyses of histology and tumor size on cancer-specific death.

	Histology				Tumor size			
	Small cell carcinoma	*P*	Non small lung carcinoma	*P*	≤2 cm	*P*	>2 cm	*P*
Chemotherapy								
No vs yes	2.16 (0.94, 4.99)	0.070	0.92(0.70, 1.21)	0.568	0.79 (0.46, 1.26)	0.393	0.86 (0.63, 1.16)	0.317
Radiation/surgery								
No	Ref		Ref		Ref		Ref	
Surgery alone	0.28 (0.11, 0.76)	0.012	0.38 (0.28, 0.53)	<0.01	0.52 (0.31, 0.87)	0.013	0.33 (0.22, 0.48)	0.001
Radiation + surgery	0.85 (0.28, 2.55)	0.769	0.67 (0.47, 0.93)	0.019	0.59 (0.33, 1.07)	0.081	0.71 (0.49, 1.02)	0.065
Radiation alone	0.41 (0.18, 0.90)	0.026	0.66 (0.50, 0.88)	0.005	0.61 (0.36, 1.03)	0.064	0.63 (0.47, 0.86)	0.003

## Data Availability

The data underlying this study were obtained from open SEER databases. All relevant data are within the paper and its Supplementary Materials.

## Supplementary Materials

Supplementary Materials Table S1: risk factors of cancer cause death in prognostic nomogram with Cox regression model among patients with metachronous second primary lung cancer. S1 Figure: the screen of independent risk factors of cancer-specific death using the least absolute shrinkage and selection operator (LASSO) analysis with Cox regression model. (a) Tuning parameter selection in the LASSO model used 10-fold cross-validation via minimum criteria. (b) LASSO coefficient profiles of potential risk factors. (c) The area under the receiver operating characteristic curve (AUC = 0.84). S2 Figure: the prognostic nomogram for predicting the 1-year and 5-year probability of cancer-specific death among metachronous SPLC by using Cox regressiong model. Total point values were independently calculated for each cause of death and then applied to the corresponding probability scale at the bottom of each. S3 Figure: decision curve analysis for cancer-specific death model among metachronous SPLC by using Cox regression model. (a) 1-year probability of cancer-specific death; (b) 5-year probability of cancer-specific death. S4 Figure: calibration plot for cancer-specific death model among metachronous SPLC by using Cox regression model. S5 Figure: calibration plot for cancer-specific death model among metachronous SPLC by using competing risk model. The solid line represents equality between the predicted and observed probabilities. With the dots close to the solid line, the plots reveal excellent agreement between the nomogram-predicted probabilities and actual observations.
