# Supplementary material for: Prognostic Nomogram and Therapeutic Option of Cancer-Specific Death in the Patients with Metachronous Second Primary Lung Cancer
Source: J Oncol. 2022 Jan 18;2022:2819798. doi: 10.1155/2022/2819798 (PMC8789464; doi:10.1155/2022/2819798)
Supplement: Supplementary Materials — Table S1: risk factors of cancer cause death in prognostic nomogram with Cox regression model among patients with metachronous second primary lung cancer. S1 Figure: the screen of independent risk factors of cancer-specific death using the least absolute shrinkage and selection operator (LASSO) analysis with Cox regression model. (a) Tuning parameter selection in the LASSO model used 10-fold cross-validation via minimum criteria. (b) LASSO coefficient profiles of potential risk factors. (c) The area under the receiver operating characteristic curve (AUC = 0.84). S2 Figure: the prognostic nomogram for predicting the 1-year and 5-year probability of cancer-specific death among metachronous SPLC by using Cox regressiong model. Total point values were independently calculated for each cause of death and then applied to the corresponding probability scale at the bottom of each. S3 Figure: decision curve analysis for cancer-specific death model among metachronous SPLC by using Cox regression model. (a) 1-year probability of cancer-specific death; (b) 5-year probability of cancer-specific death. S4 Figure: calibration plot for cancer-specific death model among metachronous SPLC by using Cox regression model. S5 Figure: calibration plot for cancer-specific death model among metachronous SPLC by using competing risk model. The solid line represents equality between the predicted and observed probabilities. With the dots close to the solid line, the plots reveal excellent agreement between the nomogram-predicted probabilities and actual observations. [file 2819798.f1.doc]

| Table S1: Risk factors of cancer cause death in prognostic nomogram with Cox regression model among patients with metachronous second primary lung cancer | | |
| --- | --- | --- |
|  | Multivariate Cox regression analysis | |
|  | HR | *P* |
| **Age** |  |  |
| < 65 years vs ≥ 65 years | 1.44 (1.12, 1.86) | <0.01 |
| **Sex** |  |  |
| Female vs Male | 1.35 (1.13, 1.62) | <0.01 |
| **Marital status** |  |  |
| Unmarried vs married | 0.83 (0.69, 0.999) | 0.049 |
| **Histology (SPLC)** |  |  |
| Small cell carcinoma | Ref |  |
| Large cell carcinoma | 0.84 (0.50, 1.39) | 0.49 |
| Squamous cell carcinoma | 0.68 (0.50, 0.92) | 0.01 |
| Adenocarcinoma | 0.47 (0.64, 0.35) | <0.01 |
| Other | 0.51 (0.33, 0.78) | <0.01 |
| **AJCC N, 6th ed (SPLC)** |  |  |
| N0 | Ref |  |
| N1 | 2.02(1.34, 3.06) | <0.01 |
| N2 | 2.35(1.69, 3.27) | <0.01 |
| N3 | 2.23(1.47, 3.38) | <0.01 |
| **AJCC T, 6th ed(SPLC)** |  |  |
| T1 | Ref |  |
| T2 | 1.76(1.39, 2.22) | <0.01 |
| T3 | 2.41(1.49, 3.89) | <0.01 |
| T4 | 1.27(0.94, 1.71) | 0.12 |
| **Radiation/surgery(SPLC)** |  |  |
| No | Ref |  |
| Surgery alone | 0.28(0.21, 0.37) | <0.01 |
| Radiation+surgery | 0.56(0.41, 0.77) | <0.01 |
| Radiation | 0.55(0.43, 0.70) | <0.01 |
| Note: IPLC, initial primary lung cancer; SPLC, second primary lung cancer. | | |


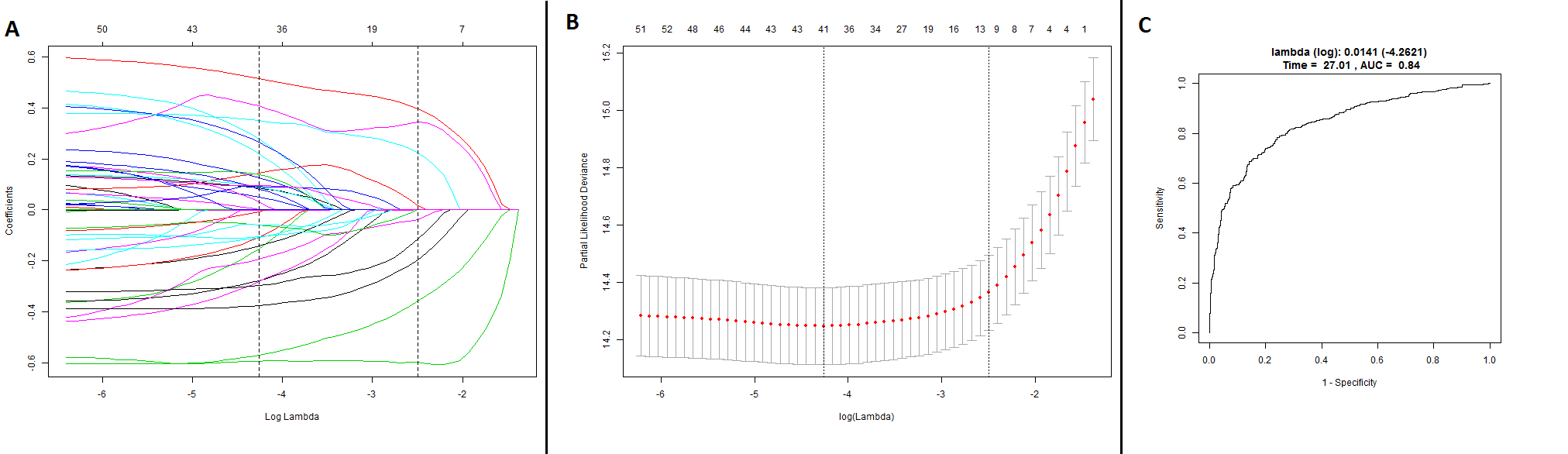


S1 Figure. The screen of independent risk factors of cancer specific death using the least absolute shrinkage and selection operator (LASSO) analysis with Cox regression model. (A) Tuning parameter selection in the LASSO model used 10-fold cross-validation via minimum criteria. (B) LASSO coefficient profiles of potential risk factors. (C) The area under the receiver operating characteristic curve (AUC = 0.84).


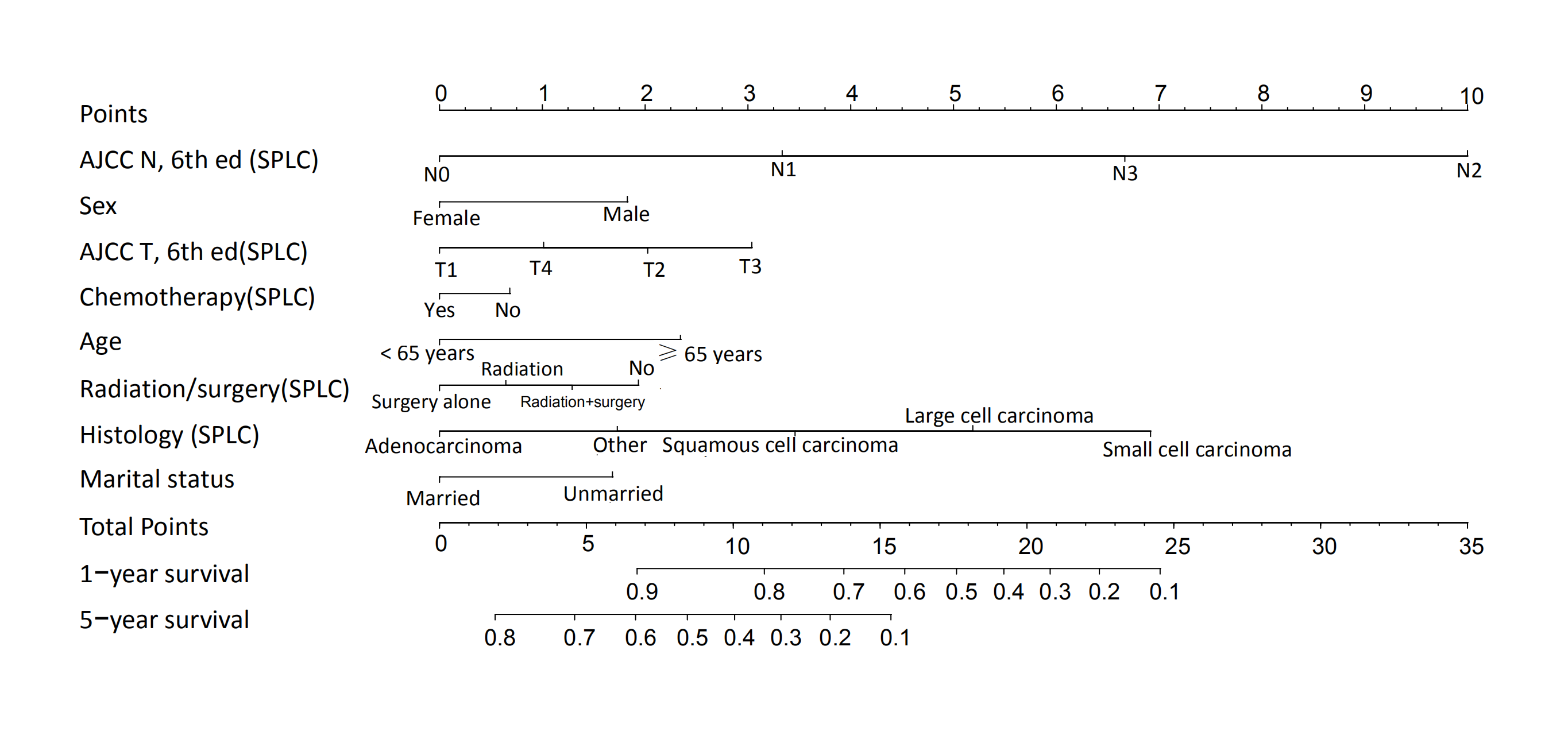


S2. Fig: The prognostic nomogram for predicting the 1-year and 5-year probability of cancer-specific death among metachronous SPLC by using Cox regressiong model. Total point values were independently calculated for each cause of death and then applied to the corresponding probability scale at the bottom of each.


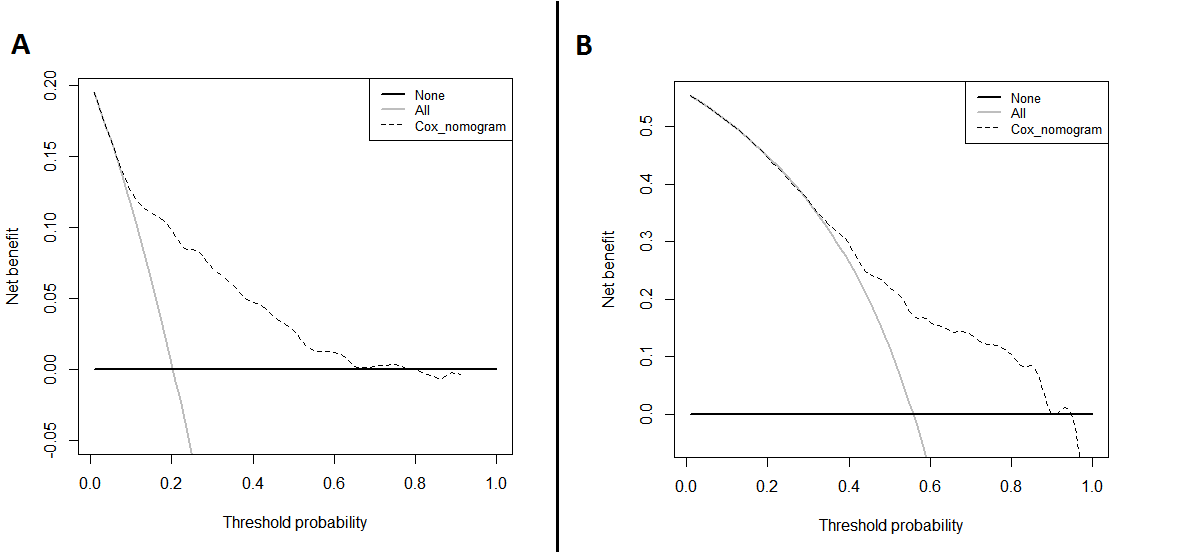


S3. Fig Decision curve analysis for cancer-specific death model among metachronous SPLC by using Cox regressiong model. (A)1-year probability of cancer-specific death;(B)5-year probability of cancer-specific death.


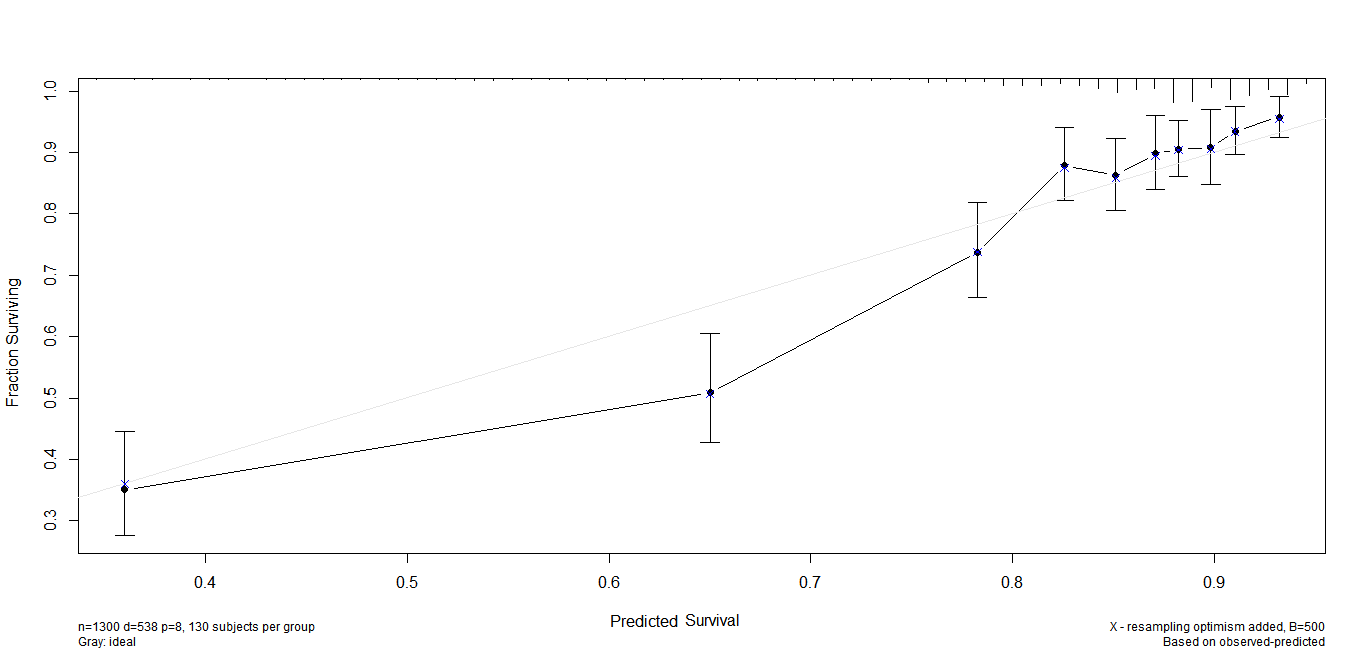


S4 Figure：Calibration plot for cancer specific death model among metachronous SPLC by using Cox regression model.


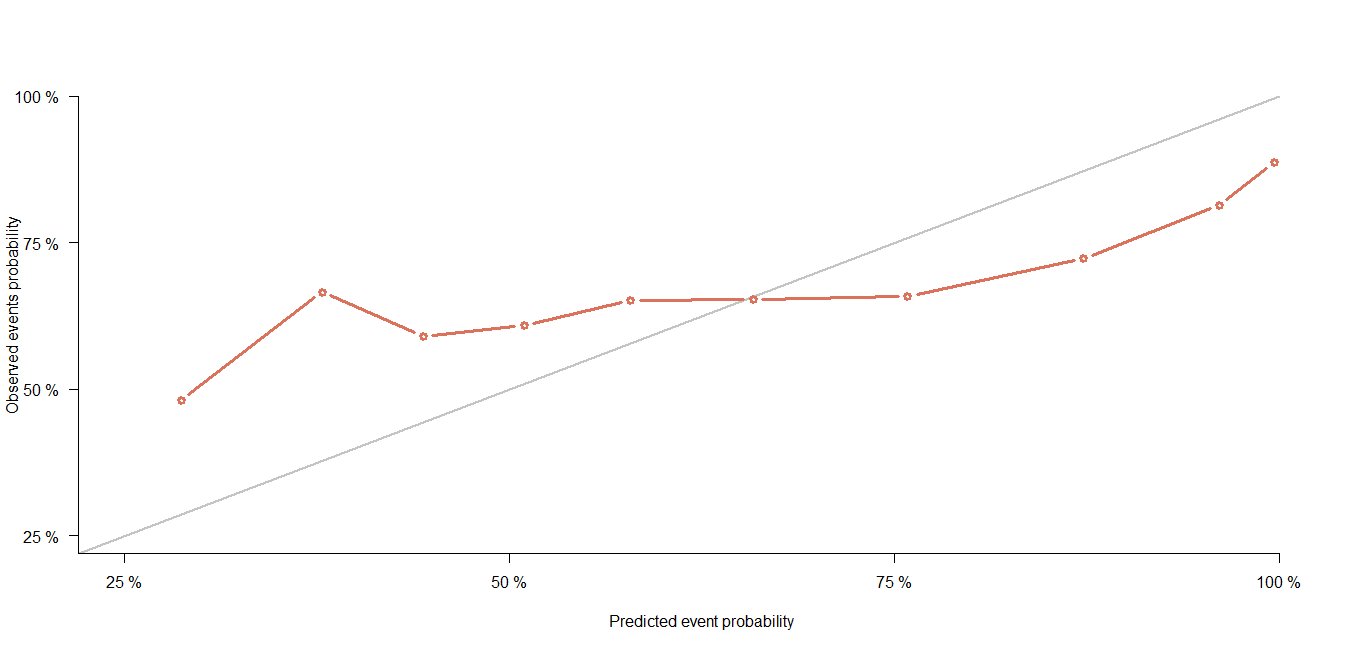


S5. Fig Calibration plot for cancer specific death model among metachronous SPLC by using competing risk model. The solid line represents equality between the predicted and observed probabilities. With the dots close to the solid line, the plots reveal excellent agreement between the nomogram-predicted probabilities and actual observations
